# Dietary Supplements Improve the Growth Performance and Carcass Yields of Indigenous Sheep in Ethiopia: A Systematic Review and Meta‐Analysis Study

**DOI:** 10.1002/vms3.70129

**Published:** 2024-11-22

**Authors:** Hussen Ebrahim, Kefyalew Alemayehu

**Affiliations:** ^1^ Department of Animal Science Woldia University Woldia Ethiopia; ^2^ Department of Animal Science Bahir Dar University Bahir Dar Ethiopia; ^3^ Ethiopian Agricultural Transformation Institute (ATI), Amhara Agricultural Transformation Center (AATC) Bahir Dar Ethiopia

**Keywords:** carcass yield, dietary supplement, growth performance, indigenous sheep

## Abstract

Despite the large number of sheep in Ethiopia, their productivity remains low, mainly due to inadequate and poor‐quality feed. Therefore, this systematic review and meta‐analysis study was conducted to evaluate the effect of dietary supplements on the growth performance and carcass yield indices of indigenous sheep in Ethiopia. This study included a total of 21 studies that used 533 sheep from 11 breeds. We used Meta‐Essentials Version 1.5 to determine the effect sizes in a mixed‐effects model at *p* < 0.05. The current meta‐analyses revealed that dietary supplements had a positive and significant effect (*p* of *Z* ≤ 0.001) on the total dry matter intake (Hedges' *g* = 6.84 g/day/head), final body weight (Hedges' *g* = 3.65 kg/head), average daily gain (Hedges' *g* = 3.59 g/head), feed conversion efficiency (Hedges' *g* = 0.72 g/g), slaughter weight (Hedges' *g* = 2.56 kg/head) and hot carcass (Hedges' *g* = 2.73 kg/head) of sheep compared to the control. The meta‐analysis suggests that sheep of different breeds and sexes that were fed supplemental diets responded differently. In addition, the subgroup analysis declared that dietary supplementation of legumes resulted in a higher magnitude of effect sizes for all response variables in sheep in comparison to sheep supplemented with concentrate and browse. We detected diverse heterogeneity across studies for all response variables that ranged between 0% and 96.65%. It can be concluded that dietary supplements had a positive and significant impact on feed efficiency, growth performance and carcass yield in indigenous sheep, particularly Afar sheep, followed by Arsi Bale and Washera sheep, in Ethiopia.

## Graphical abstract

Sheep offered legume supplements performed better compared to sheep supplemented with concentrates and browse. Afar sheep offered dietary supplements had a greater magnitude of effect size than other indigenous sheep breeds.

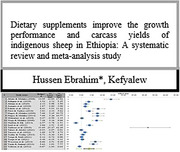

## 1 Introduction

Ethiopia had a total of 85.9 million small ruminants in 2021, of which 44.9% were sheep (FAOstat 2022). The country's contribution is estimated to be 3.6% and 9.6% of the world's and Africa's small ruminant populations, respectively (FAOstat 2022). Small ruminants are the main source of food and income, mainly in low‐income countries (Kronqvist, Kongmanila, and Wredle 2021), such as Ethiopia. The country's demand for small ruminant production is increasing due to the expansion of urbanisation, the relatively low cost of investment and resistance to disease and drought (Abraham, Gizaw, and Urge 2017). Small ruminants account for 12% of the livestock products consumed and 48% of the cash income (Taju 2018). In Ethiopia, farmers raise small ruminants primarily for income generation (Armson et al. 2020; Abdilahi et al. 2023).

The indigenous sheep constitute more than 99% of the total sheep population in Ethiopia (CSA 2021). The majority of the indigenous sheep breeds in Ethiopia are predominantly found in the pastoral and crop‐livestock production systems, which are characterised by a low‐input, low‐output method. Sheep production serves as a saving account, particularly during crop failure due to drought, flooding and other natural phenomena (Edea et al. 2010). Moreover, sheep generate income and largely benefit smallholders in the country as sources of skin, meat and manure.

However, the productivity of sheep in Ethiopia has remained low due to an inadequate feed supply, both in quality and quantity, the occurrence of disease and parasites and the low genetic potential of the animals. Natural pasture, roadside pasture, crop stubble and riverside pasture are the main constituents of sheep feed in Ethiopia (CSA 2021). Moreover, the high cost and seasonal variation in conventional feeds have constrained smallholder farmers to raise small ruminants, mainly sheep, next to cattle (Tarekegn et al. 2022). A systematic review conducted in East Africa using 54 eligible journals (81.5% from Ethiopia) indicated that disease restricted small ruminant production (Armson et al. 2020).

The role of extensive grazing as a source of sheep feed is decreasing over time due to the expansion of bushland and its conversion into other types of land, including cropland, forest land, area exclosures and unplanned towns. On the other hand, small ruminants are preferred due to their short gestation period and their potential to survive in harsh environments (Armson et al. 2020) as the effects of climate change are thought to be severe in the future. Sheep are mainly fed nutrient‐insufficient feeds, particularly during the dry season (Kenfo, Mekasha, and Tadesse 2018). Thus, supplementing sheep with high‐quality feed is the best strategy for boosting their performance and enhancing smallholder farmers' benefits (Adugna, Mekuriaw, and Asmare 2020). However, others have reported much lower values for the contribution of supplementary feeding (Hagos and Melaku 2014). This systematic review and meta‐analysis study was therefore conducted to quantify the effects of different dietary supplements on the growth performance and carcass yields of indigenous sheep in Ethiopia.

## 2 Materials and Methods

The PRISMA guidelines were used to conduct and report this systematic review and meta‐analysis study (Moher et al. 2009). However, this study did not have an established protocol, and it was not registered online.

### 2.1 Eligibility Criteria

We identified a total of 86 studies, of which 21 were included and 65 were excluded on the basis of population, interventions, comparisons, outcomes, context and study design criteria (Table 1). Scientific publications that were written in English and conducted in Ethiopia were included in the meta‐analysis. This study contains the dates searched.

**TABLE 1:** PICOCS criteria to identify, evaluate and include studies for meta‐analysis.

PICOC	Inclusion criteria	Exclusion criteria
Population	Indigenous sheep	Other species
Interventions	Any dietary supplementation	Irrelevant treatment
Comparisons	Control group (basal diet)	No control group
Outcomes	TDMI, FBW, ADG, FCE, SW, HC and CC	No related outcome
Context	Ethiopia	Other countries
Study design	Randomized complete block design	Other designs

Abbreviation: ADG, average daily gain; CC, cold carcass; FBW, final body weight; FCE, feed conversion efficiency; SW, slaughter weight; TDMI, total dry matter intake.

### 2.2 Literature Search Strategy

**FIGURE 1:**
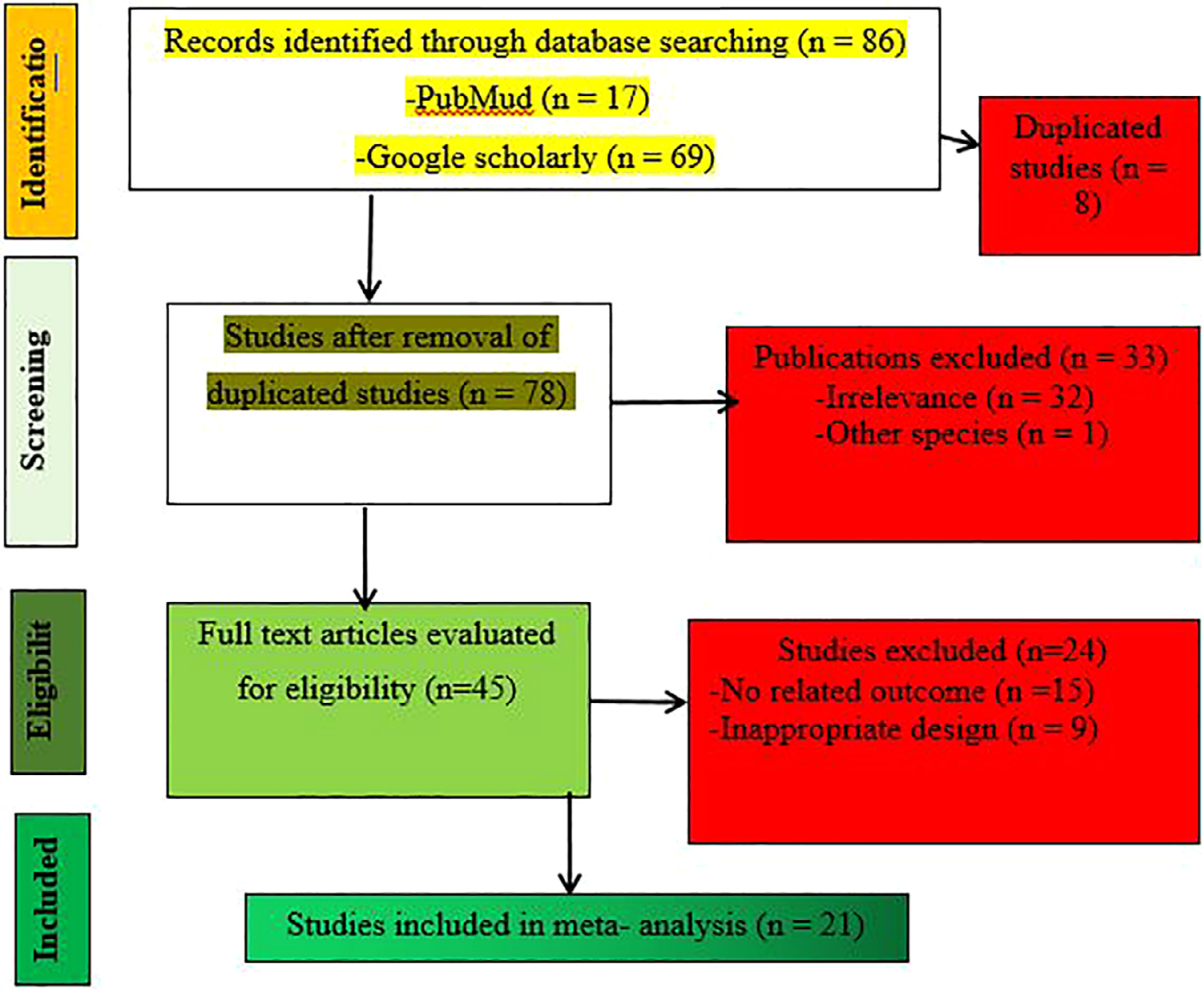
PRISMA flow chart from searching to inclusion of studies.

Electronic search strategies, such as PubMed and Google Scholar, were used to search the publications, as indicated in Table 2. The last search was recorded on 2 March 2023. The reference lists of systematically searched studies provide additional scientific papers. Figure 1 indicates the selected procedures followed during the systematic and thorough assessment of studies for the meta‐analysis study.

**TABLE 2:** Search strategies on the effects of dietary supplements on the performance of sheep in Ethiopia for a meta‐analysis study.

Database	Search strategy
PubMed	((((((Body weight)) OR (growth)) OR (reproductive performance)) OR (productive performance)) AND (((((sheep)) OR (lamb)) OR (ram)) OR (ewe)))))
Google Scholarly	Diet OR Feed OR Supplement AND Sheep AND Ethiopia

### 2.3 Study Selection

The titles and abstracts of published papers were screened after the removal of duplicates based on the eligibility criteria set out in Table 1. Then, the full texts of eligible publications were identified through a detailed evaluation. We were unable to contact the authors of the included studies to get additional information. Sheep fed conventional feeds such as hay and straw, as well as complementary feeds, were mostly considered control groups. Animals taking experimental diets of supplements were considered to be receiving treatments with no restriction on levels.

### 2.4 Data Extraction

The study was conducted without a published protocol. In this study, data were extracted based on the following: (1) authors with publication year, (2) sheep breed type, (3) number of animals, (4) type of ration, (5) experimental diet, (6) initial body weight and (7) growth and carcass response variables of the experiment. Carcass response variables were slaughter weight (SW), hot carcass (HC) and cold carcass (CC), whereas final body weight (FBW), average daily gain (ADG) and feed conversion efficiency (FCE) were considered growth response variables. The standard deviation was calculated from the standard error of the means by dividing by the square root of the number of sheep that were supplemented or not supplemented. This conversion was performed for some of the studies included. Data from studies that consisted of two or more levels of supplements were pooled for meta‐analysis.

### 2.5 Effect Size Quantification and Publication Bias Assessment

We performed a mixed‐effects model in this systematic review and meta‐analysis study to examine the effect of supplementary diets on the growth performance and carcass yield indices of indigenous sheep in Ethiopia (Rice, Higgins, and Lumley 2018; Borenstein 2019; Higgins and Thomas 2019). Hedges' *g* was considered to quantify the effect size in the current meta‐analysis study. We employed Cochran's *Q* statistics at *p* < 0.10 to evaluate heterogeneity across studies. *I* ^2^ values below 25%, 50% and 80% represented low, moderate and substantial variation among studies, respectively. Furthermore, we performed a subgroup analysis on the basis of the type of dietary supplements (legume, concentrate and browse), breed types and sex for response variables that contained ≥ eight studies (Higgins et al. 2003). We employed Orwin's Failsafe‐N to analyse the publication bias at the mean failsafe study of 0.1 (Orwin 1983). In addition, Begg and Mazumdar (1994) and Egger et al. (1997) tests at *p* < 0.10 were used to assess the presence of publication bias. The total number of potentially missing studies was checked, and the overall effect size was adjusted using the trim‐and‐fill method (Duval and Tweedie 2000). All statistical analysis procedures were performed using Meta‐Essentials Version 1.5 (Suurmond, van Rhee, and Hak 2017). Statistical significance was reported at *p* < 0.05.

## 3 Results

### 3.1 Description of the Eligible Studies

A total of 86 studies were identified through database searching, as shown in Figure 1. Table 3 shows the main characteristics of the studies included in the meta‐analysis. We used 21 eligible studies performed on indigenous sheep breeds in Ethiopia and published in English between 2008 and 2021. The present study included a total of 533 sheep, of which the Arsi Bale sheep breed contributed 30.21%, followed by Washera sheep (18.57%) and Afar sheep (12.2%). The majority of the sheep used in the present study were male (87.24%). A total of 18, 21, 20, 13, 8, 9 and 3 studies were used in the meta‐analysis to evaluate the total dry matter intake (TDMI), FBW, ADG, FCE, SW, HC and CC of sheep in Ethiopia, respectively. Of these, eight studies were conducted to evaluate the effect of different forms of browse supplements (Yeheyis et al. 2012; Yasin and Animut 2014; Mekuriaw et al. 2017; Seid, Tsega, and Demisse 2021; Talore and Abebe 2021; Tsega, Seid, and Demisse 2021; Tassew, Tolera, and Urge 2022). Three studies (Belay and Tesfaye 2014; Hagos and Melaku 2014; Woyessa, Tolera, and Diba 2014) evaluated the effect of legumes. More than half of the studies included in this systematic review and meta‐analysis were carried out on the effects of different concentrates.

**TABLE 3:** Main characteristics of the studies included in the meta‐analysis.

No.	Authors' name	Breed type	Sex	*N*	Type of ration	Experimental diet	IBW (means ± SD kg)	Response variable/s	Duration of experiment
1	Mekuriaw et al. (2017)	Washera	Male	24	Barley straw	NSC	23.46 ± 2.17	TDMI, FBW, ADG, and FCE	90 days
2	Yeheyis et al. (2012)	Washera	Male	30	NPH and CM	Sweet blue lupin	21 ± 1.38	TDMI, FBW, ADG, and FCE	69 days
3	Seid, Tsega, and Demisse (2021)	Arsi Bale	Female	24	DGH	CM	20.7 ± 1.50	TDMI, FBW, ADG, FCE, SW, HC, and CC	NA
4	Tsega, Seid, and Demisse (2021)	Arsi Bale	Male	35	Lentil straw	Concentrate	20.8 ± 1.5	TDMI, FBW, ADG, FCE, SW, HC, and CC	NA
5	Tsega, Seid, and Demisse (2021)	Arsi Bale	Male	32	TMR	Bagasse	19.8 ± 2.4	TDMI, FBW, ADG, FCE, SW, HC, and CC	NA
6	Talore and Abebe (2021)	Doyogena	Male	24	Taro tuber and WB	RGH	23.90 ± 1.15	TDMI, FBW, ADG, FCE, SW, and HC	90 days
7	Yasin and Animut (2014)	Afar	Male	25	NPH	CSM and PJ	17.24 ± 1.76	TDMI, FBW, ADG, FCE, SW, and HC	84 days
8	Tassew, Tolera, and Urge (2022)	Arsi Bale	Male	35	FOH	Vetch	21.4 ± 0.6	TDMI, FBW, ADG, FCE, SW, and HC	97 days
9	Tesfay and Tesfay (2013)	Highland	Male	20	Wheat straw	LL and CM	16.9 ± 1.62	TDMI, FBW, ADG, and FCE	NA
10	Ftiwi and Tadess (2018)	Local	Male	25	WB and teff Straw	SSC	19 ± 1.7	TDMI, FBW, ADG, and FCE	90 days
11	Hagos and Melaku (2009)	Afar	Male	20	Teff straw	CM	18.2 ± 1.76	TDMI, FBW, ADG, FCE, SW, and HC	90 days
12	Nega and Melaku (2009)	Farta	Male	25	NPH	Rice bran and NSM	19.23 ± 0.28	TDMI, FBW, ADG, and FCE	90 days
13	Adugna, Mekuriaw, and Asmare (2020)	Gumuz	Male	20	FMS, NSC, and LBLH	Urea treatment	21.6 ± 1.31	TDMI, FBW, ADG, FCE, SW, and HC	90 days
14	Asmare et al. (2016)	Washera	Male	25	CM	NPH and DGH	19.4 ± 1.89	TDMI, FBW, ADG, and FCE	NA
15	Abate and Melaku (2009)	Arsi Bale	Male	35	Urea‐treated barley straw	Vetch and lucerne	16.4 ± 0.56	TDMI, FBW, ADG, and FCE	90 days
16	Eshete, Gizaw, and Seifu (2012)	Menze		20	NPH and CM	Tossign	18 ± 0.62	TDMI, FBW, ADG, FCE, SW, HC, and CC	90 days
17	Tadesse, Kechero, and Tolemariam (2018)	Bonga	Female	24	Hay, concentrate and tree foliage	Wood ash	23.42 ± 0.08	FBW, ADG, and FCE	10 days
18	Nurfeta and Eik (2014)	Local	Male	30	RGH	Enset corm	16.97 ± 1.13	FBW, ADG and FCE	63 days
19	Alemu, Tegegne, and Mekuriaw (2020)	Washera	Male	20	FMS, NSC, and LBLH	Urea treatment	21.13 ± 1.77	TDMI, FBW, ADG, and FCE	90 days
20	Woyessa, Tolera, and Diba (2014)	Horro	Female	20	NPH	DVAL and SG	15.4 ± 0.58	TDMI, FBW, ADG, and FCE	90 days
21	Hagos and Melaku (2014)	Afar	Male	20	Teff straw	CM	18.23 ± 1.76	FBW, ADG, and FCE	NA

Abbreviations: ADG, average daily gain; CC, cold carcass; CM, concentrate mix; CSM, cottonseed meal; DGH, desho grass hay; DVAL, dried *Vernonia amygdalina* leaves; FBW, final body weight; FCE, feed conversion efficiency; FMS, finger millet straw; FOH, fodder oat hay; HC, hot carcass; IBW, initial body weight; KLO, khat left over; LBLH, lowland bamboo leaf hay; LL, *Leucaena leucocephala*; *N*, number of experimental animals; NPH, natural pasture hay; NSC, noug seed cake; NSM, noug seed meal; PJ, *Prosopis juliflora*; RGH, rhodes grass hay; SG, sorghum grain; SSC, sesame seed cake; SW, slaughter weight; TDMI, total dry matter intake; WB, wheat bran.

### 3.2 Total Dry Matter Intake

Eighteen studies evaluated the effect of dietary supplements on TDMI (see Table 4 and Figure S1). The combined effect analysis indicates that supplemented diets significantly increased the TDMI (*p *< 0.0001; Hedges' *g* = 6.84 g/head/day; 95% CI, 3.06, 10.63) of sheep. Subgroup analysis showed that the type of supplements significantly affected the TDMI, with sheep supplemented with legume (Hedges' *g* = 9.29 g/head/day; 95% CI, 5.42, 13.16) taking more feed than those supplemented with concentrate (Hedges' *g* = 8.84 g/head/day; 95% CI, 2.58, 15.09) and browse (Hedges' *g* = 4.37 g/head/day; 95% CI, 0.49, 9.22). Subgroup analysis results also showed that breed type significantly affected the TDMI, with Afar sheep (Hedges' *g* = 9.55 g/head/day; 95% CI, 5.31, 13.79) taking more feed than Washera sheep (Hedges' *g* = 9.28 g/head/day; 95% CI, 0.96, 19.52), Arsi Bale sheep (Hedges' *g* = 5.67 g/head/day; 95% CI, −0.89, 12.23) and local sheep (Hedges' *g* = 5.67 g/head/day; 95% CI, −0.89, 12.23) after removing Farta, Horro, Doyogena, Gumuz and Menze sheep that were reported by only one study. Female sheep (Hedges' *g* = 8.43 g/head/day; 95% CI, 2.81, 14.04) that were exposed to supplemented diets took more feed than male sheep (Hedges' *g* = 6.60 g/head/day; 95% CI, 2.67, 10.53). A substantial heterogeneity was evident across the 18 studies (*p* of *Q*‐statistic < 0.001, *I* ^2^ = 96.65) used to assess the effect of supplemented diets on the TDMI of indigenous sheep in Ethiopia. Similarly, the subgroup analysis indicated that there was substantial heterogeneity among studies for each subgroup. However, moderate heterogeneity was found among studies that used legume supplements (*p* of *Q*‐statistic = 0.091, *I* ^2^ = 64.95) and Arsi Bale sheep (*p* of *Q*‐statistic = 0.052, *I* ^2^ = 73.52).

**TABLE 4:** Effect of supplemented diets on TDMI in Ethiopian indigenous sheep.

Pooled/combined effect	NS	Hedges' *g*	CI	*I* ^2^ (%)	Hete. *p* value	*Z* value	*p* of *Z* value
Combined	18	6.84	3.06, 10.63	96.65	< 0.001	3.81	< 0.001
Diet type							
Concentrate	8	8.84	2.58, 15.09	97.13	< 0.001		
Browse	8	4.37	−0.49, 9.22	96.04	< 0.001		
Legume	2	9.29	5.42, 13.16	64.95	0.091		
Breed type							
Arsi Bale	5	5.67	−0.89, 12.23	96.79	< 0.001		
Afar	2	9.55	5.31, 13.79	73.52	0.052		
Washera	4	9.28	−0.96, 19.52	97.76	< 0.001		
Local	2	2.67	−12.61, 17.95	98.72	< 0.001		
Sex							
Male	16	6.60	2.67, 10.53	96.73	< 0.001		
Female	2	8.43	2.81, 14.04	85.76	0.008		

Abbreviations: CI, confidence interval; NS, number of included studies in the meta‐analysis.

### 3.3 Final Body Weight

Table 5 and Figure S3 illustrate 21 studies reporting FBW in which the combined effect estimate showed that supplemented sheep had a significant increment in FBW of 3.65 kg/head (95% CI, 1.33–5.97 kg/head). Subgroup analysis showed that the type of supplemented diets significantly affected the FBW, with sheep supplemented with legumes (Hedges' *g* = 7.11 kg/head; 95% CI, 6.08, 8.13) having a greater weight than those supplemented with concentrate (Hedges' *g* = 5.52 kg/head; 95% CI, 1.24, 9.79) and browse (Hedges' *g* = 1.23 kg/head; 95% CI, −0.25, 2.7). Subgroup analysis results also showed that breed type significantly affected the FBW, with Afar sheep (Hedges' *g* = 6.72 kg/head; 95% CI, 5.30, 8.15) having a greater final weight than Washera sheep (Hedges' *g* = 0.81 kg/head; 95% CI, −0.59, 2.22), Arsi Bale sheep (Hedges' *g* = 3.32 kg/head; 95% CI, −1.24, 7.87) and local sheep (Hedges' *g* = 0.52 kg/head; 95% CI, −0.89, 12.23), after removing Farta, Horro, Doyogena, Gumuz, Highland and Menze sheep that were reported by only one study. Female sheep (Hedges' *g* = 11.48 kg/head; 95% CI, −4.44, 27.4) that were exposed to supplemented diets had a greater FBW than male sheep (Hedges' *g* = 3.00 kg/head; 95% CI, 1.17, 4.84). Both the combined effect analysis and the subgroup analysis detected substantial heterogeneity (*p* of *Q*‐statistic < 0.001, *I* ^2^ = 86.91–97.76) across the 21 studies that reported the FBW of indigenous sheep in Ethiopia. However, low heterogeneity was found among studies that used legume supplements (*p* of *Q*‐statistic = 0.561, *I* ^2^ = 0) and Arsi Bale sheep (*p* of *Q*‐statistic = 0.305, *I* ^2^ = 15.81).

**TABLE 5:** Effect of supplemented diets on FBW in Ethiopian indigenous sheep.

Pooled/combined effect	NS	Hedges' *g*	CI	*I* ^2^ (%)	Hete. *p* value	*Z* value	*p* of *Z* value
Combined	21	3.65	1.33, 5.97	94.66	< 0.001	3.29	0.001
Diet type							
Concentrate	11	5.52	1.24, 9.79	95.71	< 0.001		
Browse	8	1.23	−0.25, 2.7	89.88	< 0.001		
Legume	2	7.11	6.08, 8.13	0	0.561		
Breed type							
Arsi Bale	5	3.32	−1.24, 7.87	94.94	< 0.001		
Afar	3	6.72	5.30, 8.15	15.81	0.305		
Washera	4	0.81	−0.59, 2.22	86.91	< 0.001		
Local	2	0.52	−5.82, 7.01	97.76	< 0.001		
Sex							
Male	18	3.00	1.17, 4.84	94.23	< 0.001		
Female	3	11.48	−4.44, 27.4	94.77	< 0.001		

Abbreviations: CI, confidence interval; NS, number of included studies in the meta‐analysis.

### 3.4 Average Daily Gain

The meta‐analysis of 20 eligible studies indicated that dietary supplements significantly improved the ADG of indigenous sheep in Ethiopia compared to the control groups (Hedge's *g* = 3.59 kg/head; 95% CI, 1.22, 5.96), with significant heterogeneity (*I* ^2^ = 94.92%) among studies (Table 6; Figure S4). Subgroup analysis disaggregated on the basis of types of dietary supplements revealed that sheep fed legumes had a greater effect size (Hedges' *g* = 7.33 kg/head; 95% CI, 6.36, 8.29) than sheep supplemented with concentrate (Hedges' *g* = 3.95 kg/head; 95% CI, 0.11, 7.8) and browse (Hedges' *g* = 2.25 kg/head; 95% CI, −0.23, 4.74). Subgroup analysis also revealed that sheep breed types had a positive and significant impact on ADG in indigenous sheep, with the Afar breed (Hedges' *g* = 8.01 kg/head; 95% CI, 7.72, 8.30) scoring greater effect sizes than the Arsi Bale breed (Hedges' *g* = 2.06 kg/head; 95% CI, 0.73, 3.39), Washera breed (Hedges' *g* = 1.98 kg/head; 95% CI, −‐0.63, 4.58) and local breed (Hedges' *g* = 0.53 kg/head; 95% CI, −10.43, 11.49), after excluding breeds (Farta, Horro, Doyogena, Gumuz, Menze, Bonga and Highland) that had only one study in their strata. Female indigenous sheep (Hedges' *g* = 4.02 kg/head; 95% CI, 1.07, 6.97) offered dietary supplements had a statistically higher ADG than male sheep (Hedges' *g* = 3.54 kg/head; 95% CI, 0.90, 6.18). Although there was evidence for substantial heterogeneity across studies for each subgroup ranging between (*I* ^2^ = 82.52% and 98.53%), studies performed on legume supplements and Afar breed had no heterogeneity (*I* ^2^ = 0%).

**TABLE 6:** Effect of supplemented diets on ADG in Ethiopian indigenous sheep.

Pooled/combined effect	NS	Hedges' *g*	CI	*I* ^2^ (%)	Hete. *p* value	*Z* value	*p* of *Z* value
Combined	20	3.59	1.22, 5.96	94.92	< 0.001	3.17	0.001
Diet type							
Concentrate	11	3.95	0.11, 7.8	95.03	< 0.001		
Browse	7	2.25	−0.23, 4.74	95.04	< 0.001		
Legume	2	7.33	6.36, 8.29	0	0.593		
Breed type							
Arsi Bale	4	2.06	0.73, 3.39	82.52	0.001		
Afar	3	8.01	7.72, 8.30	0	0.964		
Washera	4	1.98	−0.63, 4.58	95.13	< 0.001		
Local	2	0.53	−10.43, 11.49	98.53	< 0.001		
Sex							
Male	17	3.54	0.90, 6.18	95.31	< 0.001		
Female	3	4.02	1.07, 6.97	89.63	< 0.001		

Abbreviations: CI, confidence interval; NS, number of included studies in the meta‐analysis.

### 3.5 Feed Conversion Efficiency

The combined analysis of 13 studies revealed that dietary supplements significantly improved the FCE of indigenous sheep in Ethiopia in comparison with controls (Hedges' *g* = 0.72 g weight gain/g feed consumed; 95% CI, 0.49, 0.96) (Table 7; Figure S2). The subgroup analysis showed that indigenous sheep offered legume supplements (Hedges' *g* = 0.96 g/g; 95% CI, 0.02, 1.9) had higher effect sizes of FCE than sheep offered concentrate (Hedges' *g* = 0.95 g/g; 95% CI, 0.65, 1.25) and browse supplements (Hedges' *g* = 0.46 g/g; 95% CI, 0.43, 0.50). Similarly, Afar sheep fed supplementary diets had enhanced FCE (Hedges' *g* = 1.45 g/g; 95% CI, 1.45, 1.45) compared to Arsi Bale (Hedges' *g* = 0.48 g/g; 95% CI, 0.39, 0.57), Washera (Hedges' *g* = 0.44 g/g; 95% CI, 0.40, 0.48) and local breeds (Hedges' *g* = 0.72 g/g; 95% CI, 0.67, 0.77). We detected no heterogeneity across the 13 studies (*I* ^2^ = 0%) used for the combined effect analysis and most of the subgroup analysis. However, we found low heterogeneity for sheep offered legume supplements (*p* of *Q* = 0.208, *I* ^2^ = 36.95%).

**TABLE 7:** Effect of supplemented diets on FCE in Ethiopian indigenous sheep.

Pooled/combined effect	NP	Hedges' *g*	CI	*I* ^2^ (%)	Hete. *p* value	*Z* value	*p* of *Z* value
Combined	13	0.72	0.49, 0.96	0	0.807	6.83	< 0.001
Diet type							
Concentrate	6	0.95	0.65, 1.25	0	0.743		
Browse	5	0.46	0.43, 0.50	0	1.000		
Legume	2	0.96	0.02, 1.9	36.95	0.208		
Breed type							
Arsi Bale	2	0.48	0.39, 0.57	0	0.882		
Afar	2	1.45	1.45, 1.45	0	1.000		
Washera	2	0.44	0.40, 0.48	0	0.946		
Local	2	0.72	0.67, 0.77	0	0.939		
Sex							
Male	12	0.74	0.52, 0.96	0	0.755		
Female	1	0.52	−0.54, 1.57				

Abbreviations: CI, confidence interval; NS, number of included studies in the meta‐analysis.

### 3.6 Slaughter Weight

Eight studies evaluated the effect of supplementary diets on the SW of indigenous sheep, as depicted by Table 8 and Figure S5. The combined effect analysis indicated that sheep exposed to supplementary diets were 2.56 kg (95% CI, −0.31, 5.42) more likely to have a greater SW compared to nonsupplemented animals. Indeed, there were no legume‐based studies subjected to a subgroup analysis. The subgroup analysis demonstrated that sheep offered concentrate supplements had a greater magnitude of effect sizes (Hedges' *g* = 9.59 kg/head; 95% CI, 6.93, 12.24) in comparison to sheep offered browse supplements (Hedges' *g* = 1.2 kg/head; 95% CI, 0.49, 1.92). The subgroup analysis also revealed that Afar sheep offered dietary supplements had a higher magnitude of SW effect size (Hedges' *g* = 4.71 kg/head; 95% CI, −2.67, 12.09) than Arsi Bale sheep (Hedges' *g* = 1.54 kg/head; 95% CI, 0.81, 2.26). We observed substantial heterogeneity (*p* of *Q* < 0.001, *I* ^2^ = 90.24%) across studies based on the pooled analysis. The subgroup analysis detected substantial heterogeneity between studies for Afar sheep (*p* of *Q* < 0.001, *I* ^2^ = 95.70%) and moderate heterogeneity for Arsi Bale sheep (*p* of *Q* = 0.064, *I* ^2^ = 58.71%). Likewise, the subgroup analysis based on the supplemented diet type revealed moderate and low heterogeneity for browse (*p* of *Q* = 0.05, *I* ^2^ = 70.20%) and concentrate (*p* of *Q* = 0.25, *I* ^2^ = 24.28%) supplements, respectively.

**TABLE 8:** Effect of supplemented diets on SW in Ethiopian indigenous sheep.

Pooled/combined effect	NS	Hedges' *g*	CI	*I^2^* (%)	Hete. *p* value	*Z* value	*p* of *Z* value
Combined	8	2.56	−0.31, 5.42	90.24	< 0.001	2.11	0.035
Diet type							
Browse	6	1.20	0.49, 1.92	70.20	0.005		
Concentrate	2	9.59	6.93, 12.24	24.28	0.25		
Breed type							
Arsi Bale	4	1.54	0.81, 2.26	58.71	0.064		
Afar	2	4.71	−2.67, 12.09	95.70	< 0.001		
Sex							
Male	7	2.63	−0.19, 5.44	91.15	< 0.001		
Female	1	2.44	1.25, 3.64				

Abbreviations: CI, confidence interval; NS, number of included studies in the meta‐analysis.

### 3.7 Hot Carcass

As presented in Table 9 and Figure S6, nine studies evaluated the effect of dietary supplements on HC. The combined effect analysis declared that diet supplementation had a significant improvement on the HC of sheep (Hedges' *g* = 2.73 kg/head; 95% CI, 0.41, 5.05; *p* of *Z* = 0.007) compared to the control group. The subgroup analysis demonstrated that sheep supplemented with concentrates had a greater magnitude of HC effect size (Hedges' *g* = 6.26 kg/head; 95% CI, 3.54, 8.97) in comparison to sheep offered browse supplements (Hedges' *g* = 1.26 kg/head; 95% CI, −0.22, 2.74). Similarly, the subgroup analysis revealed that Afar sheep had a higher magnitude of effect size (Hedges' *g* = 2.16 kg/head; 95% CI, −0.99, 5.31) than Arsi Bale sheep (Hedges' *g* = 1.92 kg/head; 95% CI, −0.06, 3.89) after excluding breeds (Doyogena, Gumuz and Menze) that had only one study in their strata. We inspected substantial heterogeneity across the nine studies for combined effects (*p* of *Q* < 0.001, *I* ^2^ = 92.33%) and the subgroups as well.

**TABLE 9:** Effect of supplemented diets on HC in Ethiopian indigenous sheep.

Pooled/combined effect	NP	Hedges' *g*	CI	*I^2^* (%)	Hete. *p* value	*Z* value	*p* of *Z* value
Combined	9	2.73	0.41, 5.05	92.33	< 0.001	2.72	0.007
Diet type							
Browse	6	1.26	−0.22, 2.74	87.96	< 0.001		
Concentrate	3	6.26	3.54, 8.97	81.17	0.005		
Breed type							
Arsi Bale	4	1.92	−0.06, 3.89	89.61	< 0.001		
Afar	2	2.16	−0.99, 5.31	91.35	0.001		

Abbreviations: CI, confidence interval; NS, number of included studies in the meta‐analysis.

### 3.8 Cold Carcass

We used only three studies to investigate the effect of dietary supplements on the CC of sheep in Ethiopia, as presented in Table 10 and Figure S7. The meta‐analysis study found no significant improvement in CC (Hedges' *g* = 2.12; 95% CI, −4.46, 8.70; *p* of *Z* = 0.166) in comparison to the nonsupplemented groups of animals. This analysis considered only the effect of browse supplements due to the absence of studies on the other supplements. We detected high heterogeneity (*p* of *Q* < 0.001, *I* ^2^ = 93.26%) across studies.

**TABLE 10:** Effect of supplemented diets on CC in Ethiopian indigenous sheep.

Pooled/combined effect	NP	Hedges' *g*	CI	*I* ^2^ (%)	Hete. *p* value	*Z* value	*p* of *Z* value
Combined	3	2.12	−4.46, 8.70	93.26	< 0.001	1.38	0.166
Sex							
Male	2	0.73	−0.66, 2.12	81.47	0.020		
Female	1	5.28	3.44, 7.12	—	—		

Abbreviations: CI, confidence interval; NS, number of included studies in the meta‐analysis.

### 3.9 Publication Bias Assessment

Table 11 and Figures S8–S14 present the publication bias of studies for all response variables. The Orwin's failsafe‐N analysis detected no publication bias for all response outcomes. However, Egger's and Begg's tests revealed a significant publication bias for all response variables, except CC (*p* of Egger's = 0.176; *p* of Begg's = 0.117). The trim and fill method detected potential missing studies for all response variables except for CC. The meta‐analysis to evaluate the effect of dietary supplements on TDMI, FBW, ADG, FCE, SW and HC of sheep missed eight, seven, four, two, one and three potential studies, respectively.

**TABLE 11:** Publication risk of bias assessment and potential missing studies.

Response outcomes	Fail‐safe *N*	*p* of Begg's test	*p* of Egger's test	Number of missing studies
TDMI	0	< 0.001	< 0.001	8
FBW	0	< 0.001	< 0.001	7
ADG	0	< 0.001	< 0.001	4
FCE	0	0.001	< 0.001	2
SW	0	0.013	0.001	1
HC	0	0.004	< 0.001	3
CC	0	0.117	0.176	0

Abbreviations: ADG, average daily gain; CC, cold carcass; FBW, final body weight; FCE, feed conversion efficiency; HC, hot carcass; SW, slaughter weight; TDMI, total dry matter intake.

## 4 Discussion

The present meta‐analysis revealed that dietary supplementation, particularly legumes and concentrates, significantly improved TDMI, FBW, ADG, FCE, SW and HC in indigenous sheep in Ethiopia. This could be related to the availability and utilisation of nutrients. Sweet lupin is used as a sheep feed to improve the protein values of low‐quality natural pasture hay (Yeheyis et al. 2012). Currently, locally available industrial by‐products are widely used to improve the performance of sheep, thereby enhancing the utilization of nutrients and lessening the effect of plant secondary compounds, which are commonly said to be antinutritional factors (Nurfeta 2010). Nega and Melaku (2009) noted that supplementation of sheep with concentrates enhanced the utilisation of nutrients and hence improved the performance of animals. Hagos and Melaku (2009) also confirmed that supplementation with concentrates, which are rich in nutrients, resulted in improvements in FBW and HC in Afar sheep, even though their contribution is low.

Though industrial byproducts have the potential to enhance small ruminant production, farmers are now using browse trees to feed their animals due to the exorbitant cost and inaccessibility of concentrates. Browse plants are widely used by small ruminants for survival and changes in body weight. Research conducted on Washera sheep indicated that lucerne leaves could substitute for noug cake (Mekuriaw et al. 2017). Moringa leaves are rich in protein, minerals and vitamins and are recommended for use in sheep production to boost the benefits for smallholder farmers (Gebregiorgis, Negesse, and Nurfeta 2012). According to the study of Adem and Misbah (2021), *Vernonia amygdalina* improved the body weight gain of Somali goats, thereby increasing FCE. Mekoya et al. (2009) indicated that the inclusion of *Susbania susban* increased body weight by up to 30% by outweighing concentrate. Abergelle goats supplemented with *Acacia saligna* had higher body weight and carcass weight compared to nonsupplemented goats (Belay and Tesfaye 2014). The inclusion of Vernonia leaves and ground sorghum grain in the diet improved the body weight of Horro lambs (Woyessa, Tolera, and Diba 2014). Yasin and Animut (2014) suggested that supplementing cotton seed cake or a mixture of cotton seed cake and ground *Prosopis juliflora* appeared to be a better biological strategy than feeding hay alone to Afar yearling rams. *Ficus thonningii* leaf resulted in increased body weight gain in goats due to high intake (Balehegn, Eik, and Tesfay 2014). However, the farmers' perception is the main reason why browses from multipurpose trees are not widely used in sheep production (Mekuriaw et al. 2017).

This meta‐analysis study provides insight into the potential of dietary supplements, particularly legumes and concentrates, to improve the growth and carcass yields of indigenous sheep (especially Afar sheep) in Ethiopia. However, the current results should be used with caution due to variations in study season and agroecology. Furthermore, the study only used published results, which could result in publication bias. Therefore, an original experiment is highly recommended to provide substantial evidence.

## 5 Conclusion

The present meta‐analysis results revealed that dietary supplements improved feed intake and efficiency, growth performance and carcass yield indices in indigenous sheep in Ethiopia, except for cold carcass yield. Subgroup analysis demonstrated that sheep offered grain legume supplements had a higher magnitude in all response variables compared to sheep supplemented with concentrates and browse. The present study also indicated that Afar sheep, followed by Arsi Bale and Washer sheep offered dietary supplements, had a greater magnitude of effect size than other indigenous sheep breeds. The results showed that female sheep offered supplementary diets had better performance indices than male sheep. Moreover, the meta‐analysis found diverse heterogeneity across studies for all response variables that ranged between 0% and 96.65%. Therefore, additional meta‐analysis specific to a breed and dietary supplement is needed to provide more robust evidence.

## Author Contributions

**Hussen Ebrahim**: visualization, data extraction, data analysis, data interpretation, draft writing, editing, final write‐up. **Kefyalew Alemayehu**: visualization, editing, evaluation, validation.

## Ethics Statement

The authors confirm that the journal's ethical policies, as stated on the journal's author guideline page, have been adhered to. Because this is a review article with no original study data, no ethical approval was necessary.

## Conflicts of Interest

The authors declare no conflicts of interest.

### Peer Review

The peer review history for this article is available at https://publons.com/publon/10.1002/vms3.70129.

## Supporting information



Supporting information

## Data Availability

The authors have nothing to report.

## References

[vms370129-bib-0001] Abate, D. , and S. Melaku . 2009. “Effect of Supplementing Urea‐Treated Barley Straw With Lucerne or Vetch Hays on Feed Intake, Digestibility and Growth of Arsi Bale Sheep.” Tropical Animal Health and Production 41: 579–586. 18759063 10.1007/s11250-008-9227-1

[vms370129-bib-0002] Abdilahi, A. , K. Abdimahad , A. Mahamed , and A. Ali . 2023. “Study on Breeding Practices and Reproductive Performance of Black‐Head Somali Sheep Under Traditional Management System: The Case of Awbarre District, Eastern Ethiopia.” Open Journal of Animal Sciences 13: 20–33. 10.4236/ojas.2023.131002.

[vms370129-bib-0003] Abraham, H. , S. Gizaw , and M. Urge . 2017. “Milk Production Performance of Begait Goat Under Semi‐Intensive and Extensive Management in Western Tigray, North Ethiopia.” Livestock Research for Rural Development 29: 240.

[vms370129-bib-0004] Adem, K. , and F. Misbah . 2021. “ *Vernonia amygdalina* and *Catha edulis* Leaves as Cheap Feed Source and Effect on Growth Performance of Somali Goat at East Harargha, Ethiopia.” Livestock Research for Rural Development 33: 138.

[vms370129-bib-0005] Adugna, B. , Y. Mekuriaw , and B. Asmare . 2020. “Evaluation of Untreated and Urea Molasses–Treated Finger Millet (*Eleusine coracana*) Straw and Lowland Bamboo (*Oxytenanthera abyssinica*) Leaf Hay on Nutritive Values and the Performance of Gumuz Sheep in Ethiopia.” Tropical Animal Health and Production 52: 347–355. 31352552 10.1007/s11250-019-02024-8

[vms370129-bib-0006] Alemu, D. , F. Tegegne , and Y. Mekuriaw . 2020. “Comparative Evaluation of Effective Microbe–and Urea Molasses–Treated Finger Millet (*Eleusine coracana*) Straw on Nutritive Values and Growth Performance of Washera Sheep in Northwestern Ethiopia.” Tropical Animal Health and Production 52: 123–129. 31267342 10.1007/s11250-019-01986-z

[vms370129-bib-0007] Armson, B. , A. B. Ekiri , R. Alafiatayo , and A. J. Cook . 2020. “Small Ruminant Production in Tanzania, Uganda, and Ethiopia: A Systematic Review of Constraints and Potential Solutions.” Veterinary Science 8: 5. 10.3390/vetsci8010005 PMC7824089 33561077

[vms370129-bib-0008] Asmare, B. , S. Demeke , T. Tolemariam , F. Tegegne , J. Wamatu , and B. Rischkowsky . 2016. “Evaluation of Desho Grass (*Pennisetum pedicellatum*) Hay as a Basal Diet for Growing Local Sheep in Ethiopia.” Tropical Animal Health and Production 48: 801–806. 26970971 10.1007/s11250-016-1031-8

[vms370129-bib-0009] Balehegn, M. , L. O. Eik , and Y. Tesfay . 2014. “Replacing Commercial Concentrate by *Ficus thonningii* Improved Productivity of Goats in Ethiopia.” Tropical Animal Health and Production 46: 889–894. 24715205 10.1007/s11250-014-0582-9

[vms370129-bib-0010] Begg, C. B. , and M. Mazumdar . 1994. “Operating Characteristics of a Rank Correlation Test for Publication Bias.” Biometrics 50: 1088–1101. 7786990

[vms370129-bib-0011] Belay, S. , and Y. Tesfay . 2014. “Body Weight Change and Carcass Characteristics of Abergelle Goats Supplemented With Treated *Acacia saligna* (Labill) H.L.Wendi. Leaves.” In Proceedings of the 21st Annual Conference of the Ethiopian Society of Animal Production (ESAP), edited by M. Tadesse , D. Geleti , and M. Hailemariam , 66–74. Addis Ababa, Ethiopia: ESAP.

[vms370129-bib-0012] Borenstein, M. 2019. Common Mistakes in Meta‐Analysis and How to Avoid Them. New Jersy: Biostat, Inc.

[vms370129-bib-0013] CSA . 2021. Livestock and Livestock Characteristics (Private Peasant Holdings) 2020/2021 (2013 E.C.) . Addiss Ababa, Ethiopia: Central Statistical Agency.

[vms370129-bib-0014] Duval, S. , and R. Tweedie . 2000. “Trim and Fill: A Simple Funnel‐Plot–Based Method of Testing and Adjusting for Publication Bias in Meta‐Analysis.” Biometrics 56: 455–463. 10877304 10.1111/j.0006-341x.2000.00455.x

[vms370129-bib-0015] Edea, Z. , A. Haile , M. Tibbo , et al. 2010. “Morphological Characterization of Bonga and Horro Indigenous Sheep Breeds Under Smallholder Conditions in Ethiopia.” Ethiopian Journal of Animal Production 9: 117–133.

[vms370129-bib-0016] Egger, M. , G. D. Smith , M. Schneider , and C. Minder . 1997. “Bias in Meta‐Analysis Detected by a Simple, Graphical Test.” BMJ 315, no. 7109: 629–634. 9310563 10.1136/bmj.315.7109.629 PMC2127453

[vms370129-bib-0017] Eshete, T. , S. Gizaw , and E. Seifu . 2012. “Effect of Inclusion of Tossign (*Thymus serrulatus*) in Concentrate Mix Supplementation on Performance and Sensory Quality of Meat of Menz Sheep.” Tropical Animal Health and Production 45: 177–184. 22639038 10.1007/s11250-012-0189-y

[vms370129-bib-0018] FAOstat, C. 2022. “Livestock Products.” https://www.fao.org/faostat.

[vms370129-bib-0019] Ftiwi, M. , and G. Tadess . 2018. “Nutrient Intake, Digestibility and Growth Performance of Local Sheep in Western Tigray, Ethiopia.” ARC Journal of Animal and Veterinary Sciences 4, no. 3: 48–59.

[vms370129-bib-0020] Gebregiorgis, F. , T. Negesse , and A. Nurfeta . 2012. “Feed Intake and Utilization in Sheep Fed Graded Levels of Dried Moringa (Moringa stenopetala) Leaf as a Supplement to Rhodes Grass Hay.” Tropical Animal Health and Production 44: 511–517. 21786162 10.1007/s11250-011-9927-9

[vms370129-bib-0021] Hagos, T. , and S. Melaku . 2009. “Feed Intake, Digestibility, Body Weight and Carcass Parameters of Afar Rams Fed Tef (*Eragrostis tef*) Straw Supplemented With Graded Levels of Concentrate Mix.” Tropical Animal Health and Production 41: 599–606. 18777140 10.1007/s11250-008-9230-6

[vms370129-bib-0022] Hagos, T. , and S. Melaku . 2014. “Evaluating the Economic Profitability of Afar Rams Supplemented With Graded Levels of Mixtures of Protein and Energy Sources: In Case of Alamata Woreda.” In Proceedings of the 21st Annual Conference of the Ethiopian Society of Animal Production (ESAP), edited by M. Tadesse , D. Geleti , and M. Hailemariam , 86–94. Addis Ababa, Ethiopia: ESAP.

[vms370129-bib-0024] Higgins, J. P. , S. G. Thompson , J. J. Deeks , and D. G. Altman . 2003. “Measuring Inconsistency in Meta‐Analyses.” British Medical Journal 327: 557–560. 12958120 10.1136/bmj.327.7414.557 PMC192859

[vms370129-bib-0025] Higgins, J. P. T. , and J. Thomas . 2019. Cochrane Handbook for Systematic Reviews of Interventions. Edited by J. P. T. Higgins , J. Thomas , J. Chandler , M. Cumpston , T. Li , M. J. Page , and V. A. Welch . Chichester, UK: Wiley. 10.1002/14651858.ED000142 PMC10284251 31643080

[vms370129-bib-0026] Kenfo, H. , Y. Mekasha , and Y. Tadesse . 2018. “A Study on Sheep Farming Practices in Relation to Future Production Strategies in Bensa District of Southern Ethiopia.” Tropical Animal Health and Production 50: 865–874. 29318531 10.1007/s11250-017-1509-z PMC5866277

[vms370129-bib-0027] Kronqvist, C. , D. Kongmanila , and E. Wredle . 2021. “Effects of Replacing Grass With Foliage on Growth Rate and Feed Intake in Goats—A Systematic Review and Meta‐Analysis.” Animals 11: 111. 34827895 10.3390/ani11113163 PMC8614473

[vms370129-bib-0028] Mekoya, A. , S. J. Oosting , S. Fernandez‐Rivera , S. Tamminga , A. Tegegne , and A. J. Van der Zijpp . 2009. “Effect of Supplementation of Sesbania sesban on Post‐Weaning Growth Performance and Sexual Development of Menz Sheep.” Livestock Science 121: 108–116.

[vms370129-bib-0029] Mekuriaw, S. , B. Hunegnaw , A. Aamane , L. Molla , A. Yitayew , and L. Yeheyis . 2017. “Tree Lucerne (*Chamaecytisus palmensis*) Leaves Substitution of Noug (*Guizotia abyssinica*) Seed Cake as Protein Supplementation on Growth Performance of Washera Sheep in Awi Zone, Amhara Region, Ethiopia.” In 2018. Proceeding of the 10th Annual Regional Conference on Livestock Completed Research Activities, edited by M. Lakew , L. Yeheyis , and M. Alemayehu , 13–16. Agricultural Research Institute, Bahir Dar: Ethiopia.

[vms370129-bib-0030] Moher, D. , A. Liberati , J. Tetzlaff , et al. 2009. “Preferred Reporting Items for Systematic Reviews and Meta‐Analyses: The PRISMA Statement.” PLoS Medicine 6: e1000097. 19621072 10.1371/journal.pmed.1000097 PMC2707599

[vms370129-bib-0031] Nega, A. , and S. Melaku . 2009. “Feed Intake, Digestibility and Body Weight Change in Farta Sheep Fed Hay Supplemented With Rice Bran and/or Noug Seed (*Guizotia abyssinica*) Meal.” Tropical Animal Health and Production 41: 507–515. 18661246 10.1007/s11250-008-9215-5

[vms370129-bib-0032] Nurfeta, A. 2010. “Feed Intake, Digestibility, Nitrogen Utilization, and Body Weight Change of Sheep Consuming Wheat Straw Supplemented With Local Agricultural and Agro‐Industrial By‐Products.” Tropical Animal Health and Production 42: 815–824. 19882225 10.1007/s11250-009-9491-8

[vms370129-bib-0033] Nurfeta, A. , and L. O. Eik . 2014. “Assessment of Different Levels of Enset (*Ensete ventricosum*) Corm as an Energy Supplement in Sheep Fed a Basal Diet of Rhodes Grass Hay.” Tropical Animal Health and Production 46: 905–911. 24715206 10.1007/s11250-014-0583-8

[vms370129-bib-0034] Orwin, R. G. 1983. “A Fail‐Safe N for Effect Size in Meta‐Analysis.” Journal of Educational Statistics 8: 157–159.

[vms370129-bib-0035] Rice, K. , J. P. Higgins , and T. Lumley . 2018. “A Re‐Evaluation of Fixed Effect (s) Meta‐Analysis.” Journal of the Royal Statistical Society: Series A 181: 205–227.

[vms370129-bib-0036] Seid, W. , W. Tsega , and E. Demisse . 2021. “Fattening Performance of Arsi‐Bale Sheep Fed on Desho Grass Hay Supplemented With Different Concentrate Levels.” In Livestock Research Results, edited by F. Feyissa , G. Kitaw , and T. Jembere . 722–731. Addis Ababa: Ethiopian Institute of Agricultural Research.

[vms370129-bib-0037] Suurmond, R. , H. van Rhee , and T. Hak . 2017. “Introduction, Comparison, and Validation of Meta‐Essentials: a Free and Simple Tool for Meta‐Analysis.” Research Synthesis Methods 8: 537–553. 28801932 10.1002/jrsm.1260 PMC5725669

[vms370129-bib-0038] Tadesse, W. , Y. Kechero , and T. Tolemariam . 2018. “Comparison of Polyethylene Glycol and Wood Ash Extract on Feeding Value and Economic Efficiency of Mixes of High‐Tannin Feed Sources in Growing Ethiopian Bonga Lambs.” Tropical Animal Health and Production 50: 161–167. 28975512 10.1007/s11250-017-1417-2

[vms370129-bib-0039] Taju, H. 2018. “Local Sheep and Goat Reproductive Performance Managed Under Farmer Condition in Southern Ethiopia.” International Journal of Livestock Production 9: 280–285.

[vms370129-bib-0040] Talore, D. G. , and A. Abebe . 2021. “Replacement of Wheat Bran for Taro Tuber (*Colocosia esculenta*) on the Fattening Performance of Doyogena Sheep Fed on Rhodes Grass (*Chlorias gayana*) Hay, Southern Ethiopia.” Journal of Animal Sciences and Livestock Production 5: 004.

[vms370129-bib-0041] Tarekegn, A. , K. Adane , D. Amsalu , and E. Gashaw . 2022. “Effects of Feeding Faba Bean Hull, Wheat Bran and Their Mixtures on Performance of Growing Local Sheep Fed a Basal Diet of Sudan Grass Hay at Gondar Zuria, Ethiopia.” Animal Science and Biotechnologies 55, no. 1: 40–50.

[vms370129-bib-0042] Tassew, B. , A. Tolera , and M. Urge . 2022. “Effect of Different Varieties of Vetch Hay Supplementation on Carcass Characteristics of Sheep Fed a Basal Diet of Fodder Oat Hay.” American Journal of Life Sciences 10: 78–87.

[vms370129-bib-0043] Tesfay, T. , and Y. Tesfay . 2013. “Partial Replacement of Dried *Leucaena leucocephala* (Lam.) de Wit Leaves for Noug (*Guizotia abyssinica*)(L.f.) Cass. Seed Cake in the Diet of Highland Sheep Fed on Wheat Straw.” Tropical Animal Health and Production 45: 379–385. 22820996 10.1007/s11250-012-0227-9

[vms370129-bib-0044] Tsega, W. , W. Seid , E. Demisse , and A. Kasahun . 2021. Growth Performance and Carcass Yield of ArsiBale Sheep Fed on Sugarcane Bagasse Based Ration. Results of Livestock Research Completed in 2020, Addis Ababa, Ethiopia.

[vms370129-bib-0045] Woyessa, F. , A. Tolera , and D. Diba . 2014. “Feed Intake and Growth Performance and Economic Returns of Horro Lambs Fed Natural Pasture Hay Supplemented With Graded Levels of Dried *Vernonia amygdalina* Leaves and Sorghum Grain Mixture.” In Proceedings of the 21st Annual Conference of the Ethiopian Society of Animal Production (ESAP), edited by M. Tadess , D. Geleti , and M. Hailemariam , 75–85. Addis Ababa, Ethiopia: ESAP.

[vms370129-bib-0046] Yasin, M. , and G. Animut . 2014. “Replacing Cottonseed Meal With Ground Prosopis Juliflora Pods; Effect on Intake, Weight Gain and Carcass Parameters of Afar Sheep Fed Pasture Hay Basal Diet.” Tropical Animal Health and Production 46: 1079–1085. 24823899 10.1007/s11250-014-0615-4

[vms370129-bib-0047] Yeheyis, L. , C. Kijora , F. Tegegne , and K. J. Peters . 2012. “Sweet Blue Lupin (*Lupinus angustifolius* L.) Seed as a Substitute for Concentrate Mix Supplement in the Diets of Yearling Washera Rams Fed on Natural Pasture Hay as Basal Diet in Ethiopia.” Tropical Animal Health and Production 44: 1255–1261. 22228539 10.1007/s11250-011-0066-0

