# Supplementary material for: Dietary Supplements Improve the Growth Performance and Carcass Yields of Indigenous Sheep in Ethiopia: A Systematic Review and Meta‐Analysis Study
Source: Vet Med Sci. 2024 Nov 22;11(1):e70129. doi: 10.1002/vms3.70129 (PMC11582474; doi:10.1002/vms3.70129)
Supplement: Supplementary file 1 — Supporting information [file VMS3-11-e70129-s001.docx]

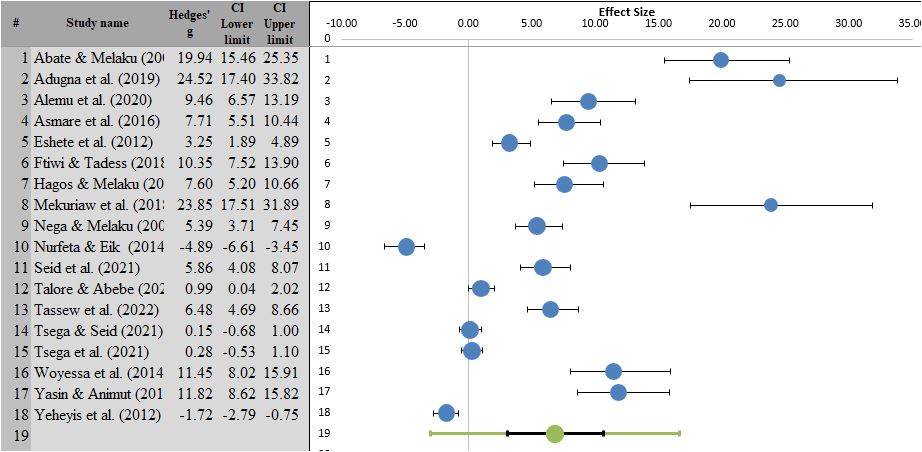


**Sup. Figure 1. Effect size and forest plot for the impact of dietary supplements on the total dry matter intake of indigenous sheep in Ethiopia.**

**
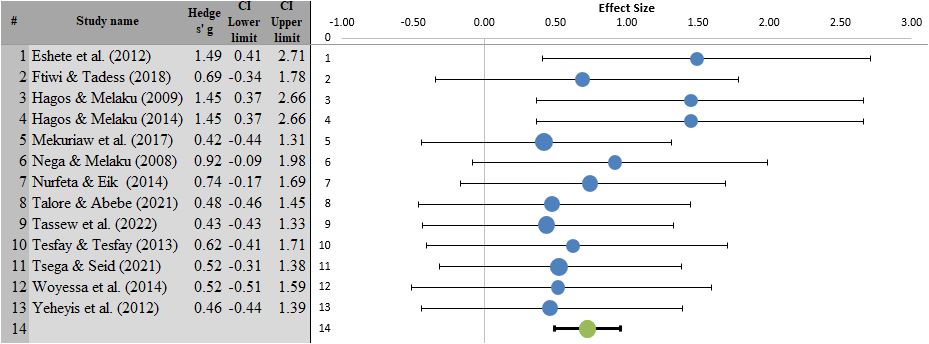
**

**Sup. Figure 2. Effect size and forest plot for the impact of dietary supplements on the feed conversion efficiency of indigenous sheep in Ethiopia.**

**
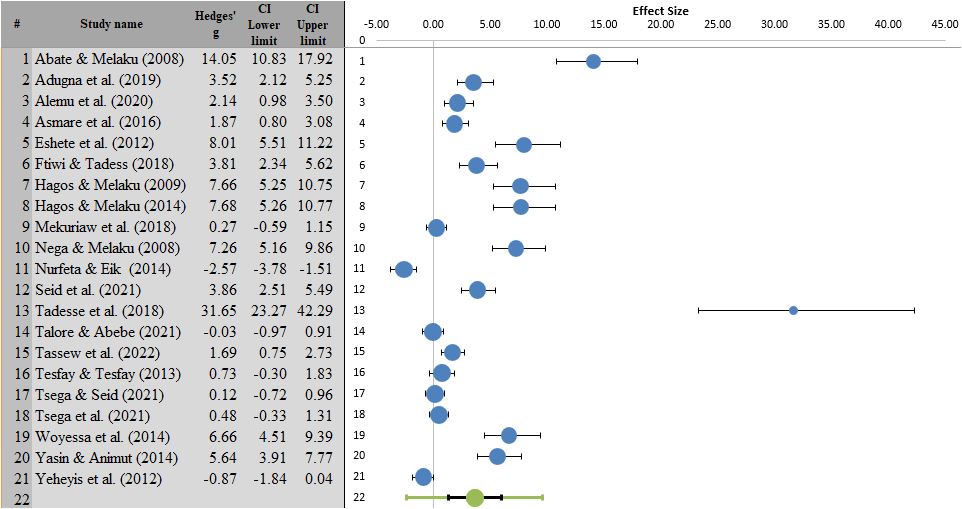
**

**Sup. Figure 3. Effect size and forest plot for the impact of dietary supplements on the final body weight of indigenous sheep in Ethiopia.**

**
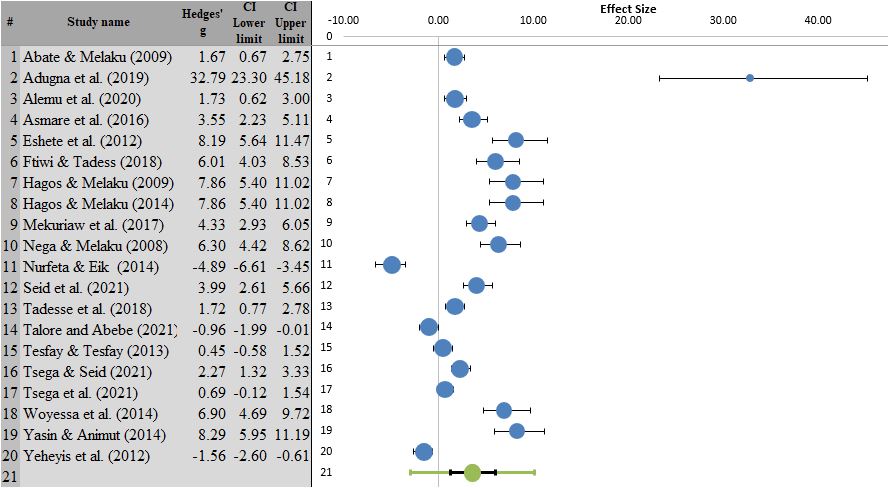
**

**Sup. Figure 4. Effect size and forest plot for the impact of dietary supplements on the average daily gain of indigenous sheep in Ethiopia.**

**
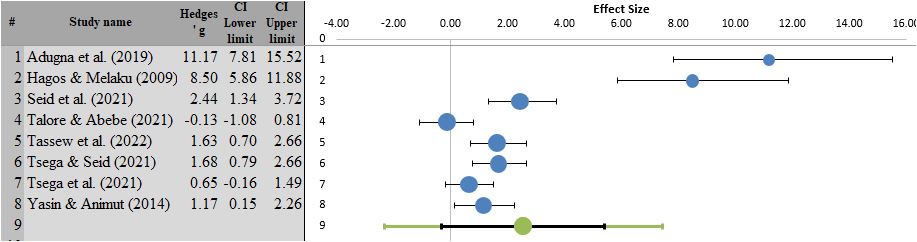
**

**Sup. Figure 5. Effect size and forest plot for the impact of dietary supplements on the slaughter weight of indigenous sheep in Ethiopia.**

**
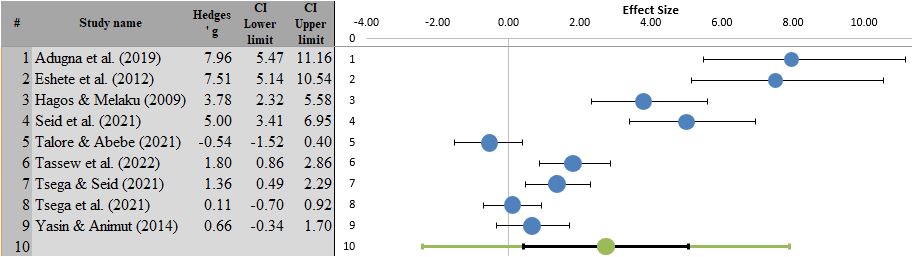
**

**Sup. Figure 6. Effect size and forest plot for the impact of dietary supplements on the hot carcass of indigenous sheep in Ethiopia.**

**
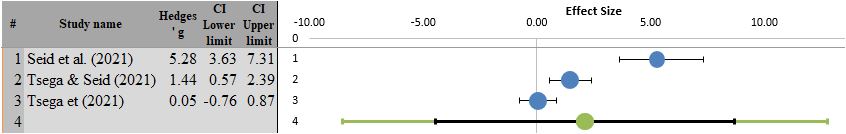
**

**Sup. Figure 7. Effect size and forest plot for the impact of dietary supplements on the cold carcass of indigenous sheep in Ethiopia.**

**
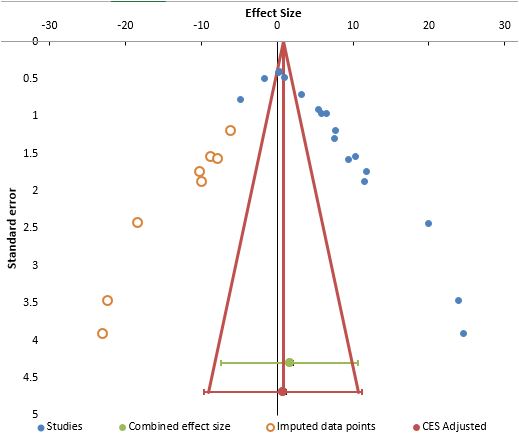
**

**Sup. Figure 8. Funnel plot for the effects of dietary supplements on the total dry matter intake of indigenous sheep in Ethiopia.**

**
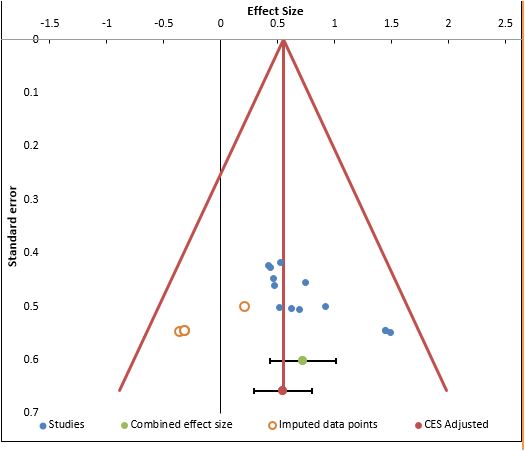
**

**Sup. Figure 9. Funnel plot for the effects of dietary supplements on the feed conversion efficiency of indigenous sheep in Ethiopia.**

**
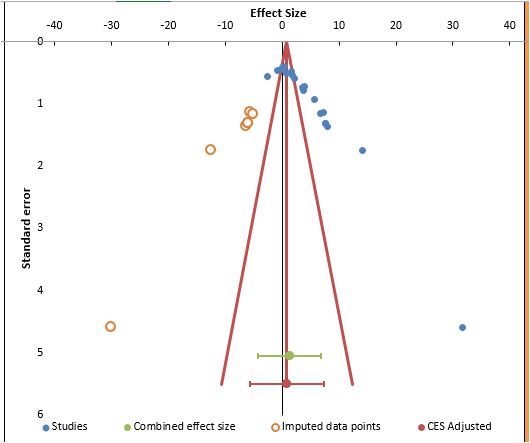
**

**Sup. Figure 10. Funnel plot for the effects of dietary supplements on the final body weight of indigenous sheep in Ethiopia.**

**
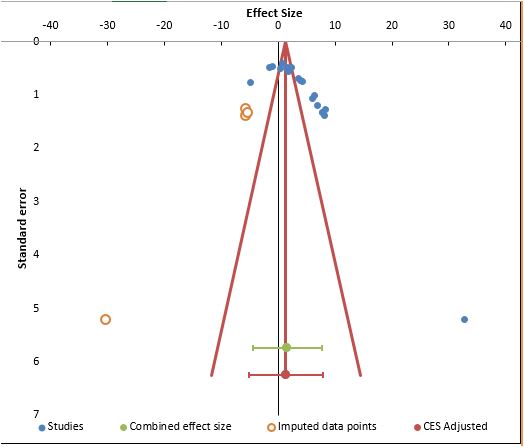
**

**Sup. Figure 11. Funnel plot for the effects of dietary supplements on the average daily gain of indigenous sheep in Ethiopia.**

**
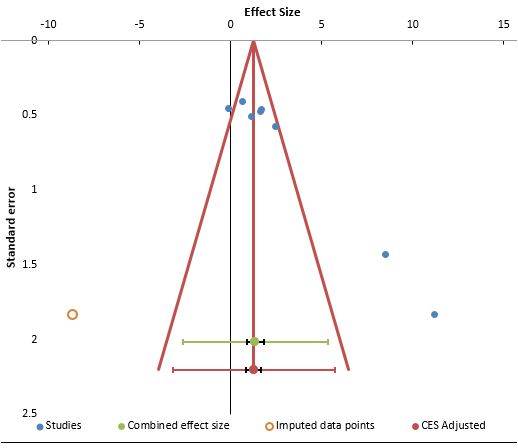
**

**Sup. Figure 12. Funnel plot for the effects of dietary supplements on the slaughter weight of indigenous sheep in Ethiopia.**

**
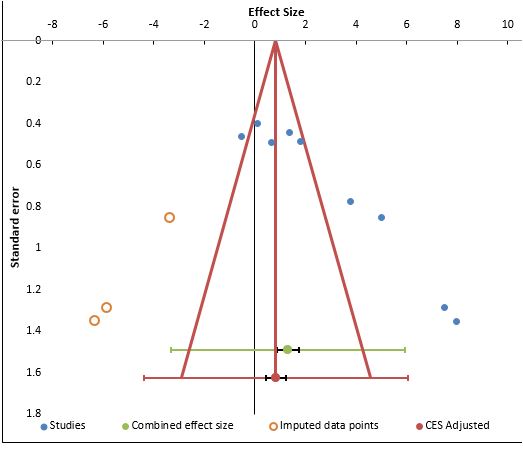
**

**Sup. Figure 13. Funnel plot for the effects of dietary supplements on the hot carcass of indigenous sheep in Ethiopia.**

**
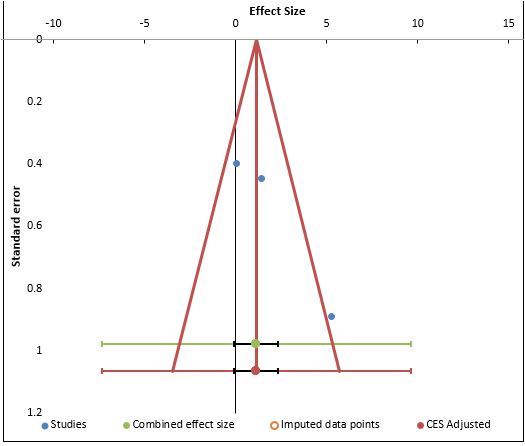
**

**Sup. Figure 14. Funnel plot for the effects of dietary supplements on the cold carcass of indigenous sheep in Ethiopia.**
